# Do post-trauma symptoms mediate the relation between neurobiological stress parameters and conduct problems in girls?

**DOI:** 10.1186/s13034-016-0129-0

**Published:** 2016-11-01

**Authors:** Kimberly A. Babel, Tijs Jambroes, Sanne Oostermeijer, Peter M. van de Ven, Arne Popma, Robert R. J. M. Vermeiren, Theo A. H. Doreleijers, Lucres M. C. Jansen

**Affiliations:** 1 Department of Child and Adolescent Psychiatry, VU University Medical Center, p/a De Bascule, P.O. Box 303, 1115 Duivendrecht, The Netherlands; 2 Department of Epidemiology and Biostatistics, VU University Medical Center, Amsterdam, The Netherlands; 3 Department of Child and Adolescent Psychiatry, Curium-Leiden University Medical Center, Leiden, The Netherlands; 4 Department of Criminal Law and Criminology, Leiden University, Leiden, The Netherlands

**Keywords:** Hypothalamic–pituitary–adrenal-axis, Autonomic nervous system, Conduct problems, Post-trauma, Girls

## Abstract

**Objective:**

Attenuated activity of stress-regulating systems has consistently been reported in boys with conduct problems. Results in studies of girls are inconsistent, which may result from the high prevalence of comorbid post-trauma symptoms. Therefore, the aim of the present study is to investigate post-trauma symptoms as a potential mediator in the relation between stress-regulation systems functioning and conduct problems in female adolescents.

**Methods:**

The sample consisted of 78 female adolescents (mean age 15.4; SD 1.1) admitted to a closed treatment institution. The diagnosis of disruptive behaviour disorder (DBD) was assessed by a structured interview—the diagnostic interview schedule for children version IV (DISC-IV). To assess post-trauma symptoms and externalizing behaviour problems, self-report questionnaires, youth self report (YSR) and the trauma symptom checklist for Children (TSCC) were used. The cortisol awakenings response (CAR) measured hypothalamic–pituitary–adrenal (HPA) axis activity, whereas autonomous nervous system (ANS) activity was assessed by heart rate (HR), pre-ejection period (PEP) and respiratory sinus arrhythmia (RSA). Independent t-tests were used to compare girls with and without DBD, while path analyses tested for the mediating role of post- trauma symptoms in the relation between stress regulating systems and externalizing behaviour.

**Results:**

Females with DBD (n = 37) reported significantly higher rates of post-trauma symptoms and externalizing behaviour problems than girls without DBD (n = 39). Path analysis found no relation between CAR and externalizing behaviour problems. With regard to ANS activity, positive direct effects on externalizing behaviour problems were present for HR (standardized β = 0.306, p = 0.020) and PEP (standardized β = −0.323, p = 0.031), though not for RSA. Furthermore, no relation—whether direct or indirect—could be determined from post-trauma symptoms.

**Conclusions:**

Present findings demonstrate that the neurobiological characteristics of female externalizing behaviour differ from males, since girls showed heightened instead of attenuated ANS activity. While the prevalence of post-trauma symptoms was high in girls with DBD, it did not mediate the relation between stress parameters and externalizing behaviour. Clinical implications and future directions are discussed.

## Background

The long-term prognosis of girls with severe conduct problems treated in mandatory closed treatment institutions is poor [1]. Adolescent girls diagnosed with a disruptive behaviour disorder (DBD) show negative outcomes in adulthood, such as early pregnancy, social isolation, personality disorders, unemployment, psychiatric co-morbidity and substance abuse [2, 3]. Current treatments are not always effective or focused on females. Understanding the specific characteristics and etiopathology of female DBD may foster specific interventions for females.

Disruptive behaviour disorder has been linked to attenuated activation of the main stress regulation systems: the Hypothalamic–pituitary–adrenal axis (HPA-axis) and the autonomic nervous system (ANS) [4]. The link between DBD and these systems is explained by the low arousal theory. According to this theory, individuals expressing conduct problems are characterized by low arousal levels, due to the lack of a physiological stress response, which may lead to individuals not fearing the negative consequences of their behaviour [5]. Alternatively, low arousal may lead to sensation-seeking behaviour in order to increase the unpleasant low arousal to normal levels [6]. Indeed, several studies in males demonstrate reduced levels of HPA-axis and ANS activity in samples with DBD or externalizing behaviour [4]. For example, Popma and colleagues [7] studied a sample of delinquent male adolescents (aged 12–14 years) and revealed that adolescents with DBD had a lower cortisol awakening response compared to controls without DBD. Regarding the ANS, a consistent finding in males with externalizing behaviour is a decreased heart rate (HR, a measure of both parasympathetic and sympathetic activity) and pre-ejection phase (PEP, which is a measure of sympathetic activity) in resting condition, and a heightened respiratory sinus arrhythmia (RSA, a measure of parasympathetic activity), also in resting condition(e.g. [8–10]). These studies provide support that low arousal, reflecting fearlessness or sensation seeking, may be a neurobiological correlate in adolescent males with externalizing behaviour.

However, research on stress regulation systems in relation to conduct problems in girls is sparse, mainly because of the low prevalence of female DBD [11]. The relatively small amount of research conducted in girls with externalizing behaviour problems provides inconclusive results. Pajer [12] studied non-referred adolescent females aged 15–17 years with conduct disorder and found diminished salivary cortisol levels in girls with DBD compared to controls, similar to the findings in boys. Likewise, Platje et al. [13] found decreased cortisol levels in girls from the general population aged 15–17 years with externalizing behaviour problems. In contrast, the study of Dorn et al. [14] found no significant associations between low arousal and conduct problem in girls aged 6–11 years, as their cortisol output was similar to those in healthy controls. Furthermore, with regard to the ANS, the relationship between DBD and low arousal in females remains disputable. A meta-analysis in children and adolescents aged from 3 to 18.5 years by Ortiz and Raine [4], suggests that low resting heart rate is diagnostically specific for both males and females with antisocial behaviour. Despite newer studies, more inconsistent findings are added to the literature regarding ANS activity and female DBD. A more recent study of Beauchaine and colleagues [15] found that aggressive girls show similar autonomic response patterns to stress as normal control girls do. Also Aults et al. [16] demonstrate that in aggressive adolescents mean age 12.4 years, from the general population, females show different autonomic reactivity than boys.

Possible explanations for the inconclusive results, besides the sparse studies of females, are large differences in sample characteristics, such as age, research population, setting, heterogeneity of quantifying conduct problems and different assessments of stress—regulation system parameters [17]. As suggested by Beauchaine [15], an important possible explanation is the presence of co-morbid internalizing disorders in female aggressive behaviour, as post-trauma psychopathology. Post-trauma psychopathology, including post-traumatic stress disorder (PTSD), has been linked to hyperresponsivity of the ANS, and this hyperresponsivity may “normalize” ANS functioning in the aggressive subgroup [15]. The inconclusive findings in literature on the relation between externalizing behaviour and functioning of stress regulation systems could therefore result from ignoring comorbid post-trauma psychopathology. Girls with conduct problems have substantially higher prevalence rates of PTSD than boys with DBD [18–20]. However, the prevalence of trauma exposure does not differ between boys and girls with conduct problems, the difference relays in the type of trauma. Females are 3–10 times more frequently the victim of sexual abuse, which is often accompanied by physical and emotional abuse; girls therefore are more often the victims of poly-traumatization [19, 20]. Hamerlynck and colleagues [21] studied a sample of detained girls aged 12–18 years, in Dutch juvenile justice institutions, and found that 21% of the girls with severe aggression also demonstrated post-traumatic stress symptoms. Moreover, a positive correlation was found between the number of traumatic experiences and extend of aggressive behaviour. This suggests that trauma exposure and subsequent post-trauma symptoms in girls are related to aggressive behaviour, a core feature of DBD. When investigating the stress-regulation system in samples diagnosed with PTSD, a common finding is decreased basal activity of stress-regulation systems, but often in combination with hyperresponsivity to stress [22]. Although acute stress causes increased activity of the HPA-axis, which results in elevated cortisol levels [23, 24], chronic or frequent stress leads to sensitization of the HPA axis. In the case of chronic stress, negative feedback mechanisms cause a shift of internal predetermined levels [25], which results in reduced physiological function at rest and hyperreactivity to stressful situations [24]. Indeed, King et al. [26] demonstrated significant lower morning saliva cortisol levels in a group of sexually abused young girls (aged 5–7 years) compared to a control group of community children. A review of PTSD in children and adolescents age ranging from 6.4 to 15.9 years demonstrated alteration in the sympathetic ANS system, which results in elevated HR in samples with PTSD [27]. El-Sheikh and Hinnant [28] revealed that girls who experienced more relational stress over time demonstrated decreased RSA while at rest and higher RSA reactivity to stress compared to boys. As such, the study confirmed that stress-regulating systems in girls may respond differently to chronic stress than those of boys.

Thus, the relation between decreased activity of the stress regulation systems and externalizing behaviour problems is well established in males; however, this relation is less clear in females and post-trauma psychopathology may influence this relation. Therefore, the present study aims to investigate the relation between the main stress-regulating systems and externalizing behaviour in girls and, subsequently, the extent to which traumatic stress symptoms mediate this relation. We hypothesize that female adolescents with conduct problems report more post-trauma symptoms and no difference will be found between their stress regulation system and that of female adolescents without conduct problems and post-trauma symptoms.

## Methods

### Participants

Female adolescents selected from a mandatory closed treatment institution for adolescents with severe behaviour problems (aged 12–18 years) in Amsterdam, the Netherlands. Placement is a result of civil law assigning them to residential care in order to receive treatment. Most frequently occurring problems include conduct problems, attention deficit disorder, disrupted personality development, drug abuse and trauma. In total, 88 girls admitted between December 2011 and February 2013, were approached to participate in this study. Of the 88 girls selected, five refused to participate, three parents disapproved of the participation of their daughter and another two were unable to participate due to early outplacements. The initial sample consisted of 78 female adolescents between 12 and 18 years old (mean age 15.4, SD 1.1). The ethnicity of the final population is as follows: 42.9% Native Dutch, 19.5% Surinamese, 13% Moroccan, 6.5% Sub-Saharan African, 5.2% Latin American, and 12.9% other ethnicity.

**Fig. 1:**
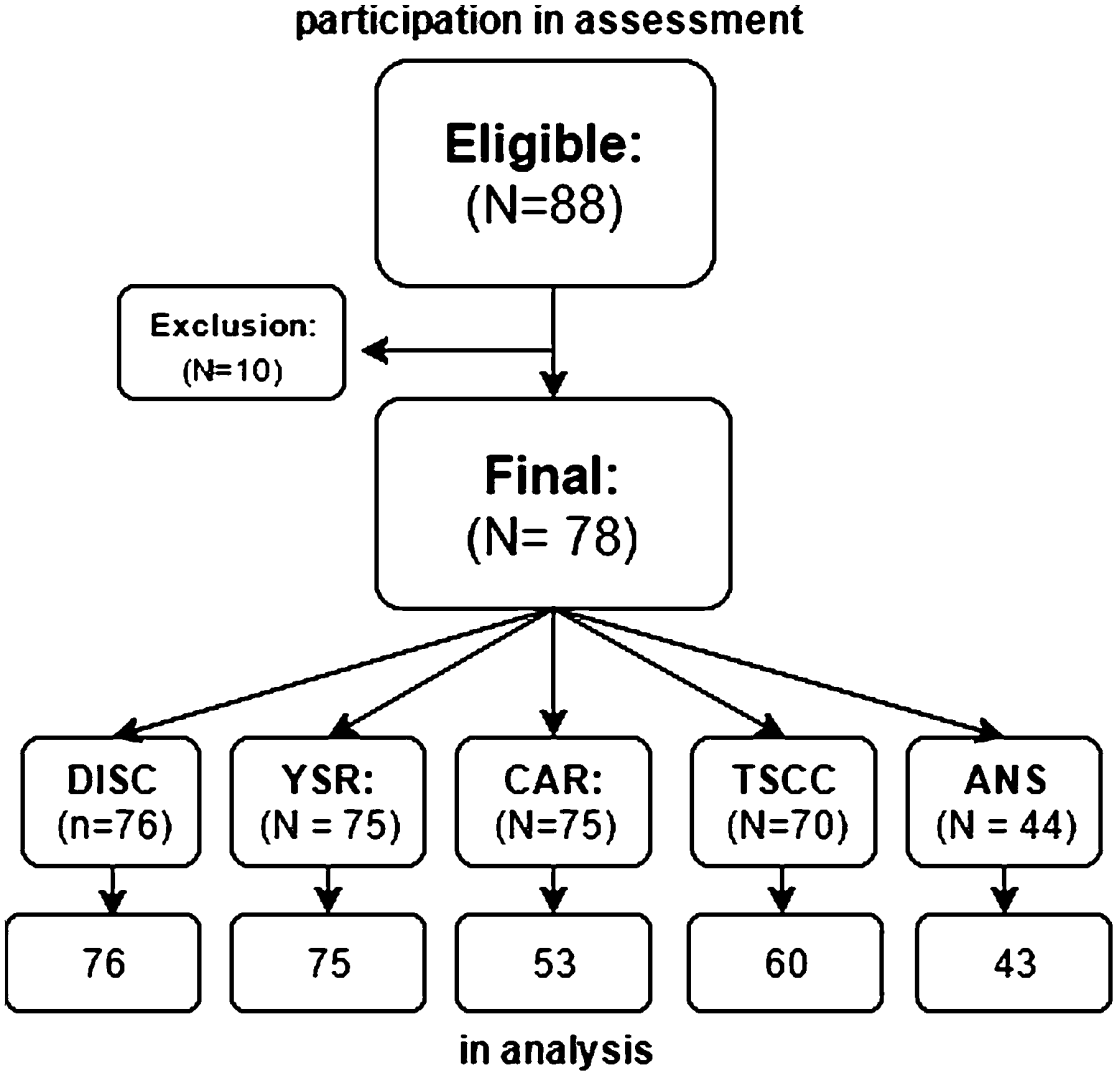
Flow chart for inclusion of participants. *DISC* Diagnostic interview schedule for children version IV, *YSR* youth self report, *CAR* cortisol awakenings response, *TSCC* trauma symptom checklist for children, *ANS* autonomous nervous system

In the analysis for HPA-axis functioning, a smaller sample was used, as three participants refused to participate in cortisol sampling, two quit during sampling and 20 samples were excluded due to meeting exclusion criteria or because of contamination during sampling. The exclusion criteria were as follows: use of medication with corticosteroids, diagnosis of a psychotic disorder, or pregnancy. One subject was using medication with corticosteroid and three were apparently pregnant during sampling. The contaminations included brushing teeth, eating between sampling time or awakening more than 15 min prior to the sampling, despite our strict instructions [29, 30]. This led to a final sample of 53 girls for the cortisol analyses. Shortly after the start of the current study, ANS measurements were added in the institution as part of a larger study. Due to the somewhat delayed start of ANS measurements, 44 girls participated in the cardiac function measurement as a correlate of ANS functioning (see flow chart of participants in Fig. 1).

### Procedure

After admission to the institution, diagnostic interviews and self-report questionnaires were completed by the admitted adolescents as part of the standard diagnostic procedures in the institution. Four weeks after admission, participants were asked to participate in the additional neurobiological measures for the current study, as placement into the institution can be considered a highly stressful experience. The four weeks allow the participants to acclimatize to the rules and daily structure in the closed treatment facility. The procedure was first explained verbally by the investigator and, after initial agreement, the girls received an additional information letter. The participating girls then signed an informed consent form. In addition, parents were informed about the study and their permission was requested for participation of their daughter. If parents agreed, they were asked to sign for informed consent. The board of the Medical Ethics Commission of the VU University Medical Center approved the project.

### Measurements

#### Disruptive behaviour disorder

Disruptive behaviour disorders were assessed using the national institute of mental health (NIMH) diagnostic interview schedule for children version IV (DISC-IV). The NIMH DISC-IV is a structured interview to asses more than 30 common child- and adolescent psychiatric diagnoses, according to the diagnostic and statistical manual of mental disorders IV (DSM-IV). The test–retest reliability on the child interview is sufficient especially for conduct disorder. Trained interviewers administered this structured interview. The participants were classified as having disruptive behaviour disorders when they fulfilled criteria for oppositional defiant disorder (ODD) and/or conduct disorder (CD), according to DSM-IV [31].

#### Externalizing behaviour disorder

To assess externalizing behaviour problems the youth self report (YSR) was used [32]. The YSR is a self-report questionnaire that measures emotional and behavioural problems. This measurement provides dimensional data of both internalizing and externalizing problems. The questionnaire consists of 112 items and can be categorized into three scales: internalizing, externalizing and neither internalizing or externalizing. The current study used only the externalizing scale. The items are scored as 0 (not true), 1 (somewhat true) and 2 (very true or often true). The raw scores are converted to T-scores, and a T-score above 65 is considered sub clinical, a score of 70 or above is considered clinically significant. Psychometric properties of this instrument have been demonstrated in prior research. The one week test–retest reliability of the YSR is r = 0.60, p < 0.05, as reported by the author, for the problem scale.

### Post-trauma symptoms

To assess trauma symptoms, the trauma symptom checklist for children (TSCC) 8–16 year was used. The TSCC is a self-report questionnaire to measure effects of childhood trauma. It consists of 54 items with six clinical scales (anxiety, anger, depression, post-traumatic stress, dissociation, and sexual concerns) and two subscales for over/underreport. The items are scored as 0 (never), 1 (sometimes), 2 (many times), 3 (almost all the time). For each scale, the raw scores are converted to T-scores; T-scores above 65 are considered indicative for the presence of that trauma symptom. A T-score above 60 on the subscales over/underreport is considered unreliable and those TSCCs were excluded from the analysis. The reliability analysis of the TSCC scales provide evidence of an a between 0.82 till 0.89 for all subscales except sexual concerns, which has an a = 0.77. Furthermore, the TSCC has a predictive validity in traumatized and non-traumatized samples. [33].

### Hypothalamus–pituitary–adrenal-axis activity

The circadian rhythm of the basal HPA-axis is reflected in cortisol levels peaking early in the morning and declining throughout the day [34]. The sharpest increase in cortisol levels is approximately 30 min after awakening, known as the cortisol awakening response (CAR). The CAR is a widely used and reliable measure for HPA axis activity [35]. The CAR requires collection of saliva at 0, 30 and 60 min post awakening. Participants were instructed not to fall asleep, eat, drink, smoke or brush their teeth during sampling. The sampling was conducted at the institution on a regular school day at 7:30, 8:00 and 8:30. The investigator woke the girls up at 7:30 and remained present for the duration of sampling to confirm that sampling was conducted correctly and to answer questions if necessary. During sampling the girls were questioned if they use conceptive and if they were on their period. 37.3% of the females used different kinds of contraceptive and 6 girls were apparently in their period during sampling. For each measurement, 0.1 ml saliva was collected in a plastic tube, using passive drooling. The samples were stored in a freezer at −20 °C until the end of the saliva collection for analysis. Analyses were performed at the Endocrinology Laboratories of the University Medical Centre Utrecht. Cortisol in saliva was measured without extraction using an in-house competitive radioimmunoassay employing a polyclonal anticortisol-antibody (K7348). [1,2-^3^H(N)]-Hydrocortisone (PerkinElmer NET396250UC) was used as a tracer. The lower limit of detection was 1.0 nmol/l. On the basis of the measurement of cortisol on t0 awakening (7:30), t1 30 min after awakening (8:00) and t2 60 min after awakening (8:30), both the *area under the curve with respect to the ground* (AUCg) and the *area under the curve with respect to increase* (AUCi) were calculated [36]. The AUCg reflects the total cortisol secretion in the first hour after awakening and is computed as follows: AUCg = ((Cortisolt1 + Cortisolt0)/2 × 30) + ((Cortisolt2 + Cortisolt1)/2 × 30)). The AUCi reflects the increase in cortisol secretion in response to awakening and is computed as follows: AUCi = ((Cortisolt1 + Cortisolt0)/2 × 30) + ((Cortisolt2 + Cortisolt1)/2 × 30) − (Cortisolt0 × (30 + 30))).

### Autonomic nerve system activity

The measurement of ANS activity was performed by measuring cardiac function with the VU Ambulatory Monitoring System (VU-AMS). This device is specifically developed for non-invasive measurement of cardiac ANS activity, and is validated for use in children [37]. Seven disposable electrodes of ‘Kendall ARBO H98SG’ with an inch of 55 mm, where placed on the chest for measurement of the Electrocardiogram (ECG) and Impedance Cardiogram (ICG). The ANS activity on the heart can be studied by examining the resting Heart Rate (HR), which is a resultant of both sympathetic and parasympathetic activity. Heightened activity of the sympathetic system causes an increased heart rate, whereas heightened activity of the parasympathetic activity causes a decreased heart rate [38, 39]. In a normal sample, the heart rate will rise during stress. The pre-ejection phase (PEP) is the time interval from the beginning of the electrical stimulation of the ventricles, the systole, to the opening of the aortic valves. It is a measure of the sympathetic activity which increases during stress, thus the shorter the PEP time interval—the higher the sympathetic activity. The respiratory sinus arrhythmia (RSA) is a measure of parasympathetic activity: the rhythmic increase in HR during inspiration and decrease during expiration [40, 41]. Normally, this variation in HR during respiration is high and is an indicator of normal parasympathetic functioning. Cardiac function was measured for 15 min during a resting condition. The participants were asked to sit in a chair and read a magazine or talk to the investigator. All of the ANS measurements were measured by a female investigator.

### Statistical analyses

Descriptive statistical analyses were performed using SPSS (version 19.0, IBM). All variables were checked in order to remove outliers due to artefacts. First, mean scores were compared between girls with and without DBD using independent t tests for all continuous variables (YSR, TSCC, and ANS and HPA measures). We performed a power analysis for the independent t test to calculate the acquired sample size, with an alpha of 0.05 and power of 0.8. Twenty-three participants per group are needed to provide reliable evidence. Subsequently, path analyses were performed for the whole group, using Mplus version 7.0 to investigate post-trauma symptoms as a possible mediator in the relation between HPA and ANS activity, and externalizing behaviour problems [42]. The six post-trauma symptom subscales were combined into a single latent variable. Per analysis, weighing of the subscales is reliant on the dependent and independent variable included in the model. Separate models were constructed for each independent HPA and ANS variable. Models included a direct effect of the independent variable on externalizing behaviour and an indirect effect via post-trauma symptoms. Standardized regression coefficients (β) are presented to quantify the strength of association between pairs of variables. Indirect mediating effects through post-trauma were tested for significance using the *model indirect statement* in Mplus. Full information maximum likelihood (FIML) was used to estimate the parameters in the model, allowing available data from participants with missing values on some of the variables to be included in the analyses. The level of significance was set at p < 0.05 for all statistical tests.

## Results

### Descriptive information for all variables distributed for disruptive behaviour disorder

Subsequently, and as presented in Table 1, the sample was divided into two groups—one comprising those with a DBD diagnosis (DBD+) and those without DBD (DBD−). Almost half the number of girls (49%) had a DBD diagnosis. Differences between the DBD+ and DBD− subgroups were analysed for all behavioural and neurobiological measures. Girls with DBD demonstrated significantly more externalizing behaviour than those without DBD [t(72) = −4.96, *p* < 0.001]. With regard to TSSC subscales, the DBD + group scored significantly higher on depression [t(57) = −2.23, *p* < 0.05], anger [t(58) = −2.74, *p* < 0.001], dissociation [t(58) = −3.18, *p* < 0.001] and sexual concerns [t(58) = −3.07, *p* < 0.001] compared to girls without DBD. No significance was noted between the DBD+ and the DBD− groups for the AUCi and AUCg, as correlates of the HPA-axis functioning, nor was there a difference for HR, PEP and RSA as correlates of the ANS functioning. There were no significant differences in age, contraceptive use and ethnicity between the DBD+ and DBD− group.

**Table 1:** Sample descriptive for all variables in the total sample and in the DBD− and DBD+ subgroups and their differences using t tests

	Total (n = 78)		DBD− (n = 39)		DBD+ (n = 37)		t	df	p
	M	SD	M	SD	M	SD			
HPA-axis (n = 53)									
AUCi (nmol/L)	334.4	262.6	332.5	274.8	343.3	257.6	−0.1	49	0.886
AUCg (nmol/L)	1100.5	297.5	1147.6	305.0	1062.6	299.3	1.0	49	0.320
ANS (n = 44)									
HR (bpm)	85.1	9.8	84.2	10.3	85.7	9.4	−0.5	41	0.628
PEP (msec)	103.7	16.5	107.7	16.5	99.0	16.0	1.7	40	0.093
RSA (msec)	101.4	49.2	102.5	48.8	102.3	51.3	0.0	41	0.990
YSR (n = 75)									
Externalizing behaviour	62.4	10.4	57.4	9.0	67.8	9.1	−5.0	72	0.001**
TSCC (n = 60)									
Anxiety	51.0	10.2	48.7	10.0	52.7	10.2	−1.5	57	0.133
Depression	52.3	9.6	49.4	7.6	54.6	10.5	−2.2	57	0.030*
Anger	48.7	8.3	45.6	7.6	51.2	8.1	−2.7	58	0.008**
Post-trauma	49.0	8.1	47.0	7.0	50.7	8.6	−1.8	58	0.074
Dissociation	50.2	9.3	46.3	7.3	53.4	9.7	−3.2	58	0.002**
Sexual concerns	53.8	17.3	46.7	10.1	59.6	19.7	−3.1	58	0.003**

*CAR* cortisol awakenings response; *YSR* youth self-report, externalizing behaviour, *DBD* disruptive behavior disorder, *AUCi* area under the curve with respect to increase, *AUCg* area under the curve with respect to the ground, *HR* heart rate, *PEP* pre ejection phase, *RSA* respiratory sinus arrhythmia

* p < 0.05, ** p < 0.01

### Path analysis cortisol awakening response

**Fig. 2:**
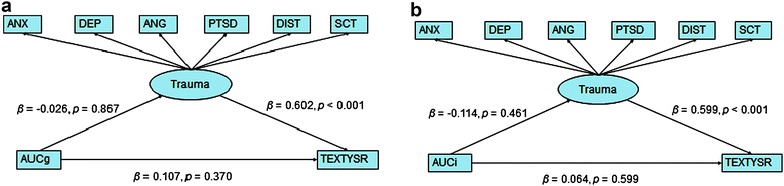
Path analysis of AUCg, AUCi and externalizing behavior and trauma as the possible mediator. *AUCg* the area under the curve with respect to the ground, *AUCi* area under the curve with respect to increase, *TEXTYSR* T-score externalizing behavior on YSR, *ANX* anxiety T-score, *DEP* depression T-score, *ANG* anger T-score, *PTSD* post-traumatic stress disorder stress symptom T-score, *DIST* dissociation T-score, *SCT* sexual concerns. T-score and βs are standardized regression coefficients

To test for the mediating role of post-trauma symptoms on the relation between the neurobiological stress regulating systems and externalizing behaviour, latent variable model analyses were performed (Fig. 2). In all models, a direct positive effect between post-trauma and externalizing behaviour was found. In Fig. 2 illustrates the latent variable models of the AUCi and AUCg, as correlates of the HPA-axis functioning in relation to externalizing behaviour and post-trauma symptoms. No significant difference was demonstrated between the correlates of HPA-axis functioning (AUCi and AUCg) and externalizing behaviour. Moreover, there was no indirect relation with post-trauma symptoms (Fig. 2).

**Fig. 3:**
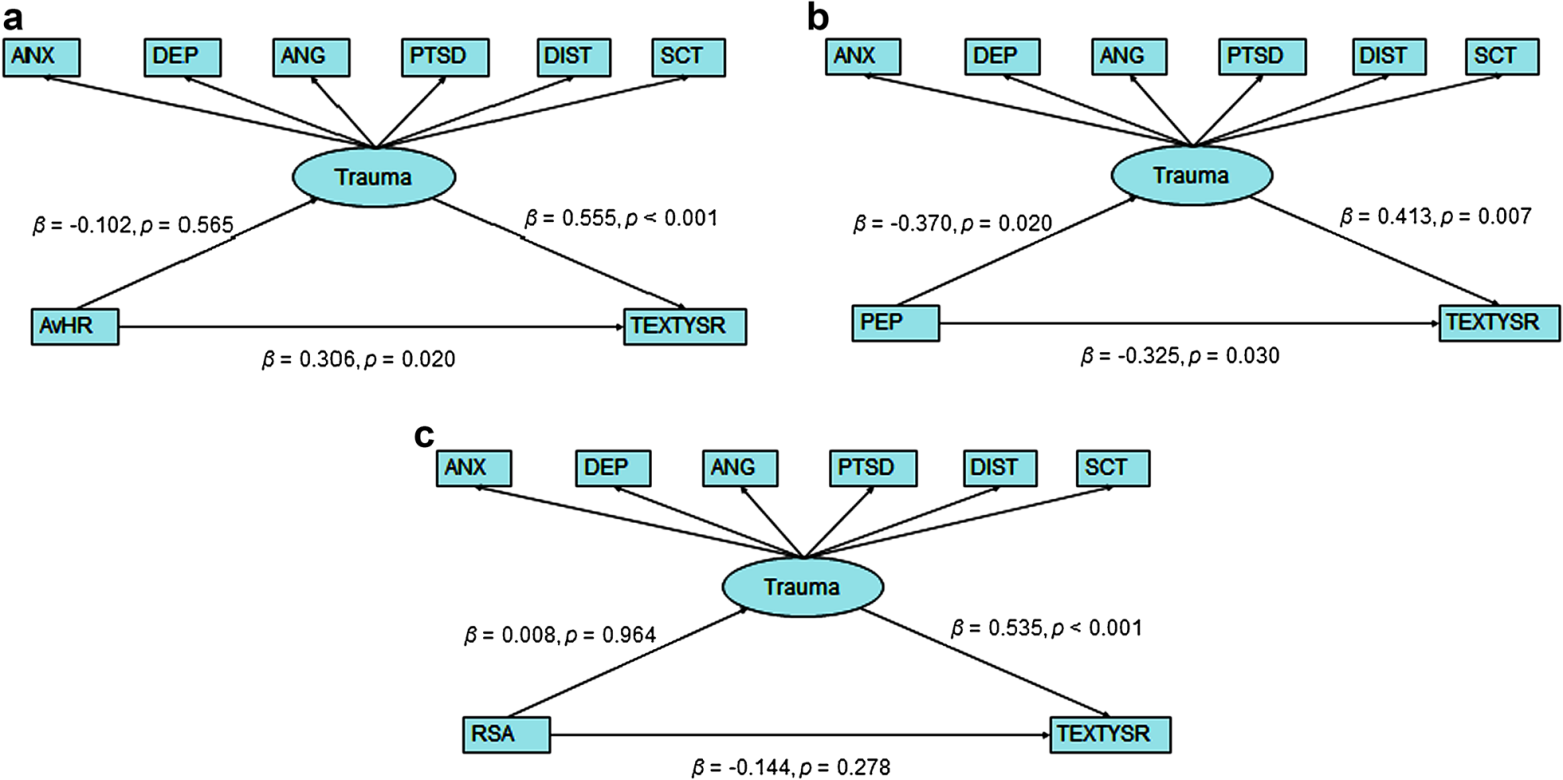
Path analysis of average heart rate, PEP, RSA and externalizing behavior with trauma as a possible mediator. *HR* average heart rate, *PEP* pre ejection phase, *RSA* respiratory sinus arrhythmia, *TEXTYSR* T-score externalizing behavior on YSR, *ANX* anxiety T-score, *DEP* depression T-score, *ANG* anger T-score, *PTSD* post-traumatic stress disorder stress symptom T-score, *DIST* dissociation T-score, *SCT* sexual concerns. T-score and βs are standardized regression coefficients

### Path analysis cardiac measurements

In Fig. 3, the latent variable models of the three ANS measures—heart rate (HR), pre ejection period (PEP) and respiratory sinus arrhythmia (RSA)—in relation to externalizing behaviour and post-trauma symptoms are presented.

The model (Fig. 3) demonstrating the HR indicates a direct positive effect between heart rate and externalizing behaviour (β = 0.135, *p* = 0.02). Likewise, a significant negative effect was found for the relation between PEP and externalizing behaviour problems (β = −0.323, p = 0.031), while the relation between RSA and externalizing behaviour was not significant. With regard to the relation between ANS correlates and post-trauma, PEP negatively correlated with post-trauma symptoms, while HR and RSA did not exhibit a significant direct relation. The relation between the ANS measures and externalizing behaviour was not influenced by any indirect effect via post-trauma symptoms (Fig. 3).

## Discussion

In this study, the relation between the two main neurobiological stress-regulating systems and conduct problems was investigated in a sample of adolescent females admitted to a mandatory closed treatment institution. Subsequently, the possible mediating role of post-trauma symptoms in this relation was tested. The findings confirmed that girls with DBD express higher rates of post-trauma symptoms than those without DBD. Furthermore, a direct positive relation between ANS activity and externalizing behaviour problems in female adolescents was found, while this was not present for HPA-axis activity. Finally, while post-trauma symptoms had a strong effect on externalizing behaviour problems, these symptoms had no mediating effect on the relation between the HPA-axis and ANS activity, or on externalizing behaviour problems in girls.

The finding that female adolescents with conduct problems express higher rates of post-trauma symptoms concurs with results from previous studies on this topic (e.g. [18–20]). Moreover, in the present study, post-trauma symptoms were positively related to externalizing symptoms, specifically the post-trauma sub dimensions: anger, depression, dissociation and sexual concerns. These results are consistent with previous findings of girls in juvenile justice institutions [21] find that at least 80% of female adolescents treated in juvenile justice institutions experienced one or more traumatic life event. Likewise, they also found a relation between traumatic life events and aggressive behaviour. These results have important clinical implications for the treatment of girls with externalizing behaviour problems in closed treatment settings. As post-trauma symptoms are a frequent finding in these girls and, as trauma exposure also relates to conduct problems, accurate assessment and specialized interventions for trauma symptoms are needed.

We did not find a decreased activity of stress regulation systems, i.e. HPA-axis activity or ANS activity, in girls with externalizing behaviour problems. Instead, externalizing behaviour symptoms correlated to an increased activity of the ANS system, expressed in a high HR and low PEP [43]. A possible explanation for this finding may be that previous research on this topic examined male samples in non-residential settings. In the meta-analysis of Raine and Ortiz [4] on the relation between ANS activity and externalizing behaviour problems, only 8 of the 40 studies included female participants. Five of the studies that included females found a relation between reduced heart rate and disruptive behaviour disorder, while all these studies used samples from the general population. The remaining three studies, which did not find any relation between ANS and externalizing behaviour problems, were performed within clinical settings [44].

Previous research that reported a decreased HPA-axis activity in girls with externalizing behaviour problems was conducted in non-clinical settings [12, 13]. The current findings indicate that low arousal may not be the underlying etiopathology for externalizing behaviour in severe clinical samples of females with DBD, such as our sample from a mandatory closed treatment institution. It is possible that the low arousal theory [5, 6] only accounts for the specific sub forms of externalizing behaviour.

In our sample, externalizing behaviour problems were associated with more comorbid post-trauma symptoms. However, post-trauma symptoms do not mediate the relation between ANS activity and externalizing problems. It is known that individuals who have experienced traumatic events react with aggressive behaviour to threat-based stimuli [20]. This form of aggression is impulsive and is accompanied by hyperarousal of the stress system. It is, furthermore, linked to early traumatic life experiences [45, 46]. This may be reflected in the current results, in which heightened activity of the ANS system and a high level of post-trauma symptoms were found. Proactive aggression, however, is non-impulsive—rather, it is calculated [47]. Core features of pro-active aggression are high levels of callous unemotional traits and hypo arousal of the stress system [48–52]. Additionally, it is linked to life-long, persistent anti-social behaviour, with an onset in early youth [53–55]. Future research investigating the low arousal theory should take these different forms of externalizing behaviour (proactive and reactive aggression), as well as post-trauma symptoms into account.

The findings in this study should be interpreted in the context of certain limitations. First, it should be noted that the sample providing ANS measurements was significantly smaller than the initial sample at the start of the study. Likewise, the exclusion of a substantial number of CAR measurements due to artefacts in the saliva collection could have influenced the results. The CAR is influenced by the menstruation cycle, anticonception use and puberty status. In this study, we reported the contraceptive use and whether the girls were menstruating at the time of sampling. However, the differences in contraceptive use or menstruation cycle vary too greatly to be taken into account. Furthermore, smoking and medication use has its influence on the cortisol and heart rate levels [41]. Acute effects of smoking on heart rate and cortisol measures were ruled out by instructing girls not to smoke within an hour before testing, data from girls who did smoke against our instructions were excluded from the analyses. However, possible long-term effect of regular smoking on heart rate and cortisol cannot be ruled out. Finally, we were not able to correct for medication use, due to imprecise collecting of medication use because we were fairly interested in cortisol containing medication.

On the other hand, t test analyses were performed with sufficient power to draw conclusions. Unfortunately, no power analysis is performed for the path analysis. Second, the study had a heterogeneous sample with an age range from 12 to 18 years. Adolescence is characterized by major behavioural and biological changes, also in the HPA-axis functioning and its relation with externalizing behaviour [13]. Future research should therefore focus on a more homogenous sample with regard to age and pubertal development, or it should perform subsequent sampling in girls during their adolescence. Lastly, the present study investigated the effect of post-trauma symptoms; however, the subscale post-traumatic stress symptoms revealed nothing significant. This can be clarified, since the post-traumatic stress symptoms measured by the TSCC was initially designed to measure sexual abuse and single traumatic events [33]. However, repetitive or complex traumatic events, such as neglect, have a higher prevalence rate and can alter a person’s psychobiological development in critical periods [56]. In addition, experiencing complex trauma can lead to complex PTSD, which differs in symptomology from PTSD [57]. Subsequently, the timing of the onset of the traumatic experience influences the HPA-axis, as recent trauma is related to increased cortisol output.

## Conclusions

Based on the present study, it can be concluded that female adolescents with DBD express high levels of comorbid post-trauma symptoms. Moreover, we found indications that the level of externalizing problems could be related to increased activity of their neurobiological stress regulating systems. In our study, these findings are different from findings in boys and result should be interpreted against the limitations (e.g. [4, 7, 8, 10, 16, 58, 55]. The findings in the current study shows that individuals with externalizing problems and high post-trauma symptoms are related to increased activation of the ANS-system. This heightened activation of the ANS-system is similar to previous findings on PTSD subject [23]. However, in this study no mediating effect was found of post-trauma symptoms on the relation between stress system activation and externalizing behaviour. One explanation may be that both findings are separate effects, or that girls with DBD and PTSD symptoms show a different, more reactive form of aggression. Future neurobiological research on externalizing behaviour problems should consider gender differences on externalizing behaviour and subsequent neurobiological mechanisms. Moreover, hyperactivity of the ANS in subgroups of girls, and perhaps also in boys, should receive specific attention, as hyperactivity of the ANS in a sample of DBD-diagnosed children proved predictive for better treatment outcome [59, 60].
